# Breaking a single hydrogen bond in the mitochondrial tRNA^Phe^‐PheRS complex leads to phenotypic pleiotropy of human disease

**DOI:** 10.1111/febs.15268

**Published:** 2020-03-18

**Authors:** Moshe Peretz, Dmitry Tworowski, Ekaterine Kartvelishvili, John Livingston, Zofia Chrzanowska‐Lightowlers, Mark Safro

**Affiliations:** ^1^ Department of Structural Biology Weizmann Institute of Science Rehovot Israel; ^2^ NHS Trust The Leeds Teaching Hospital UK; ^3^ Wellcome Centre for Mitochondrial Research Newcastle University UK

**Keywords:** human diseases, MD simulations, mitochondria, pathogenic mutations, Phenylalanyl‐tRNA synthetase, tRNA^Phe^, X‐ray structure

## Abstract

Various pathogenic variants in both mitochondrial tRNA^Phe^ and Phenylalanyl‐tRNA synthetase mitochondrial protein coding gene (FARS2) gene encoding for the human mitochondrial PheRS have been identified and associated with neurological and/or muscle‐related pathologies. An important Guanine‐34 (G34)A anticodon mutation associated with myoclonic epilepsy with ragged red fibers (MERRF) syndrome has been reported in *hmit*‐tRNA^Phe^. The majority of G34 contacts in available aaRSs‐tRNAs complexes specifically use that base as an important tRNA identity element. The network of intermolecular interactions providing its specific recognition also largely conserved. However, their conservation depends also on the invariance of the residues in the anticodon binding domain (ABD) of human mitochondrial Phenylalanyl‐tRNA synthetase (*hmit*‐PheRS). A defect in recognition of the anticodon of tRNA^Phe^ may happen not only because of G34A mutation, but also due to mutations in the ABD. Indeed, a pathogenic mutation in FARS2 has been recently reported in a 9‐year‐old female patient harboring a p.Asp364Gly mutation. Asp364 is hydrogen bonded (HB) to G34 in WT *hmit*‐PheRS. Thus, there are two pathogenic variants disrupting HB between G34 and Asp364: one is associated with G34A mutation, and the other with Asp364Gly mutation. We have measured the rates of tRNA^Phe^ aminoacylation catalyzed by WT *hmit*‐PheRS and mutant enzymes. These data ranked the residues making a HB with G34 according to their contribution to activity and the signal transduction pathway in the *hmit*‐PheRS‐tRNA^Phe^ complex. Furthermore, we carried out extensive MD simulations to reveal the interdomain contact topology on the dynamic trajectories of the complex, and gaining insight into the structural and dynamic integrity effects of *hmit*‐PheRS complexed with tRNA^Phe^.

**Database:**

Structural data are available in PDB database under the accession number(s): 3CMQ, 3TUP, 5MGH, 5MGV.

## Graphical abstract

Mutation analysis of the hydrogen bonded (HB) network between Guanine‐34 (G34) of tRNA^Phe^ and human mitochondrial Phenylalanyl‐tRNA synthetase (*hmit*‐PheRS) demonstrates that loss of HBs with Asp364Gly mutant leads to a complete enzyme inactivation. Mutation is associated with progressive paraparesis. Mutated variant G34A of tRNA^Phe^ is also accompanied by loss of H‐bond with Asp364, and significant inactivation of *hmit*‐PheRS. This mutation, however, is associated with the myoclonic epilepsy with ragged red fibers syndrome.

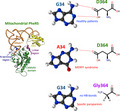

**Abbreviations**
A35: Adenine‐35
aaRS: aminoacyl‐tRNA synthetase
ABD: anticodon binding domain
DARS2: Aspartyl‐tRNA synthetase mitochondrial protein coding gene
ENM: elastic network models
FARS2: Phenylalanyl‐tRNA synthetase mitochondrial protein coding gene
G34: Guanine‐34
*hmit*‐PheRS: human mitochondrial Phenylalanyl‐tRNA synthetase
MD: molecular dynamic
MERRF: myoclonic epilepsy with ragged red fibers
mit‐aaRSs: mitochondrially localized aaRSs

## Introduction

A critical step, which determines the accuracy of protein biosynthesis and normal operation of correctly assembled macromolecules, is the delivery of the cognate amino acids to the aminoacyl‐tRNA synthetases (aaRSs). The aaRSs are therefore arguably among the most important components of the biosynthetic machinery within the cell, since they covalently attach appropriate amino acids to the corresponding nucleic acid adaptor molecules tRNA, which ensures the fidelity of translation of the genetic code [1].

The human cell contains two independent genomes both of which need to have the encoded information translated into proteins, namely the nuclear (n‐) and mitochondrial (*mit‐*)DNA. The *mit‐*DNA is a smaller genome that is present in multicopy within the mitochondrial network and encodes a different set of genes to those in the nucleus. These include 13 open reading frames for proteins within the oxidative phosphorylation complexes, the 22 tRNAs needed to translate these open reading frames, and two ribosomal RNA species [2]. With the exception of GIyRS and LysRS, cytosolic and mitochondrial aaRSs are encoding by different nuclear genes, while in mammalian cells they are encoding by the same genes [3]. In other cases, however, eukaryotic *mit*‐aaRSs exhibit higher homology to bacterial enzymes than to cytosolic counterparts from the same organism [4].

Mitochondrially localized aaRSs (*mit*‐aaRS) have been of considerable clinical interest since mutations have been identified that cause distinctive disease phenotypes dependent on the particular *mit*‐aaRS. The last two decades have accumulated both clinical and diagnostic data, indicating that mutations in either the substrate *mit*‐tRNA or in the mitochondrial aaRS can lead to diverse presentations of mitochondrial disease [5, 6, 7, 8, 9, 10, 11, 12, 13, 14, 15, 16, 17, 18, 19]. More than 200 point mutations have been identified in mitochondrial genes, the vast majority of which are associated with diverse forms of neurological diseases [20, 21, 22]. Interestingly, despite accounting for only 5–10% of the *mit*‐DNA over half of the detected mutations occurred in tRNA genes and are responsible for the majority of mitochondrial diseases [22]. Pathogenic variants have been reported in 17 of the 19 mitochondrial aaRSs genes [17]. Although these mutations cause a variety of clinical presentations, many with neurological involvement [8, 16, 23], the initial reports described very specific, restricted symptoms that were associated with a particular *mit*‐aaRS. For example, the first of these mutations to be identified was in Aspartyl‐tRNA synthetase mitochondrial protein coding gene and presented with characteristic leukoencephalopathy with brain stem and spinal cord involvement, and elevated lactate [24]. In contrast, mutations in *mit*‐ArgRS and *mit*‐TrpRS result in intellectual disability [25]. Symptoms associated with mutations in other *mit*‐aaRSs include hypertrophic cardiomyopathy, epileptic encephalopathy, and liver disease [5, 6, 25]. An increasing number of patients demonstrate that clinical manifestation, disease burden, and the patterns of which tissue is affected can be indicative of a mutation in a particular *mit*‐aaRS, permitting clinicians to predict with a high probability the affected *mit*‐aaRS from the symptoms alone [6, 19]. Since the function of the *mit*‐aaRS is the same in all tissues, and the loss of quality control in mitochondrial protein synthesis would be expected to reduce oxidative phosphorylation and therefore ATP levels in all tissues, it makes these different patterns of tissue‐specific expression particularly intriguing. The molecular consequences of the individual mutations in *mit*‐aaRS could alter their 3D structures and/ or enzymatic activities; however, the relationship between these changes and the resulting clinical manifestations remains largely unclear. As a result, mitochondrial aaRSs, *mit*‐tRNA genes, and their products have emerged as essential molecular targets for comprehensive biochemical and genetic screening across different human populations.

**Fig. 1:**
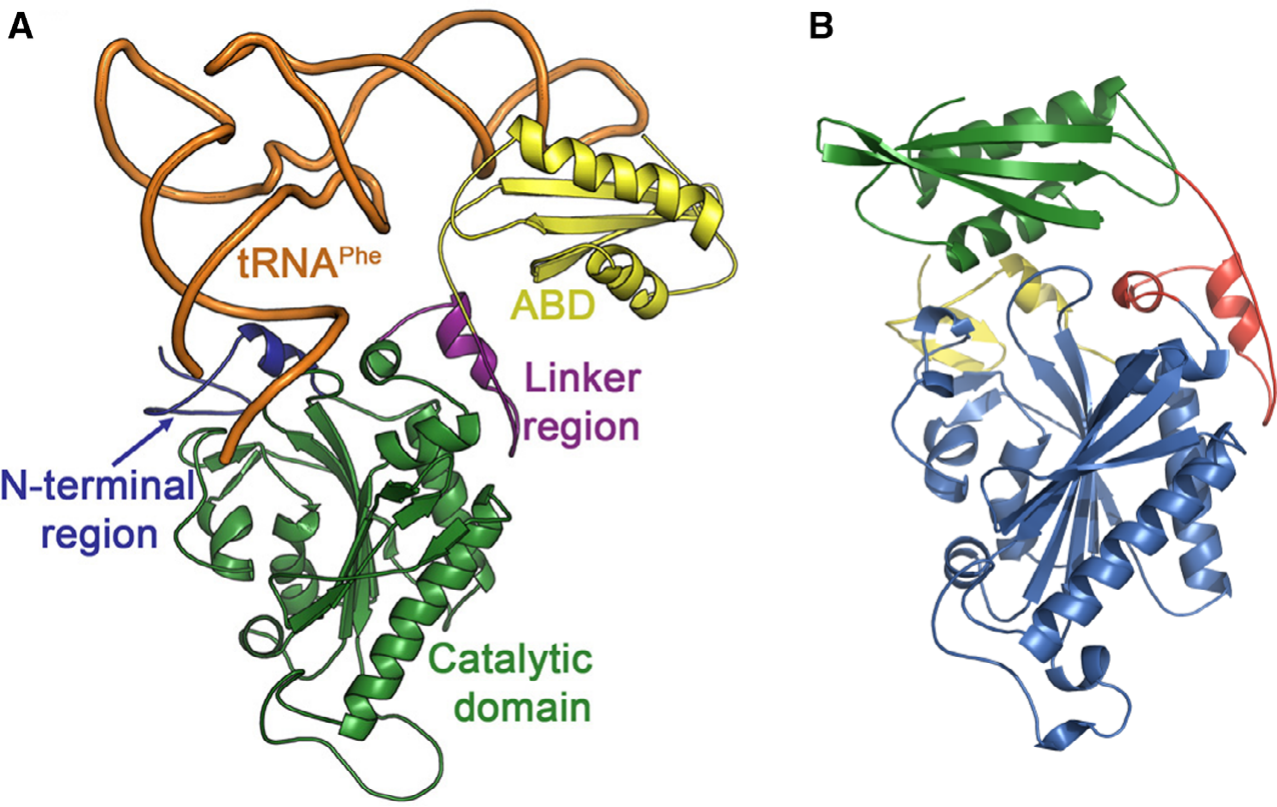
Overall crystal structure of *hmit*‐PheRS in complex with tRNA^Phe^ (open, ‘active’ conformation). (A) Ribbon representation of the *hmit‐*PheRS–tRNA^Phe^ complex. The N‐terminal region, the catalytic domain (CD), the linker region, and the ABD are colored blue, green, pink, and yellow, respectively (PDB Code 3TUP); (B) the WT crystal structure of *hmit*‐PheRS (close ‘inactive’ conformation). PDB Code 3CMQ. Figure 1A reproduced from Ref. [28]. Panels were prepared using program pymol [49].

**Fig. 2:**
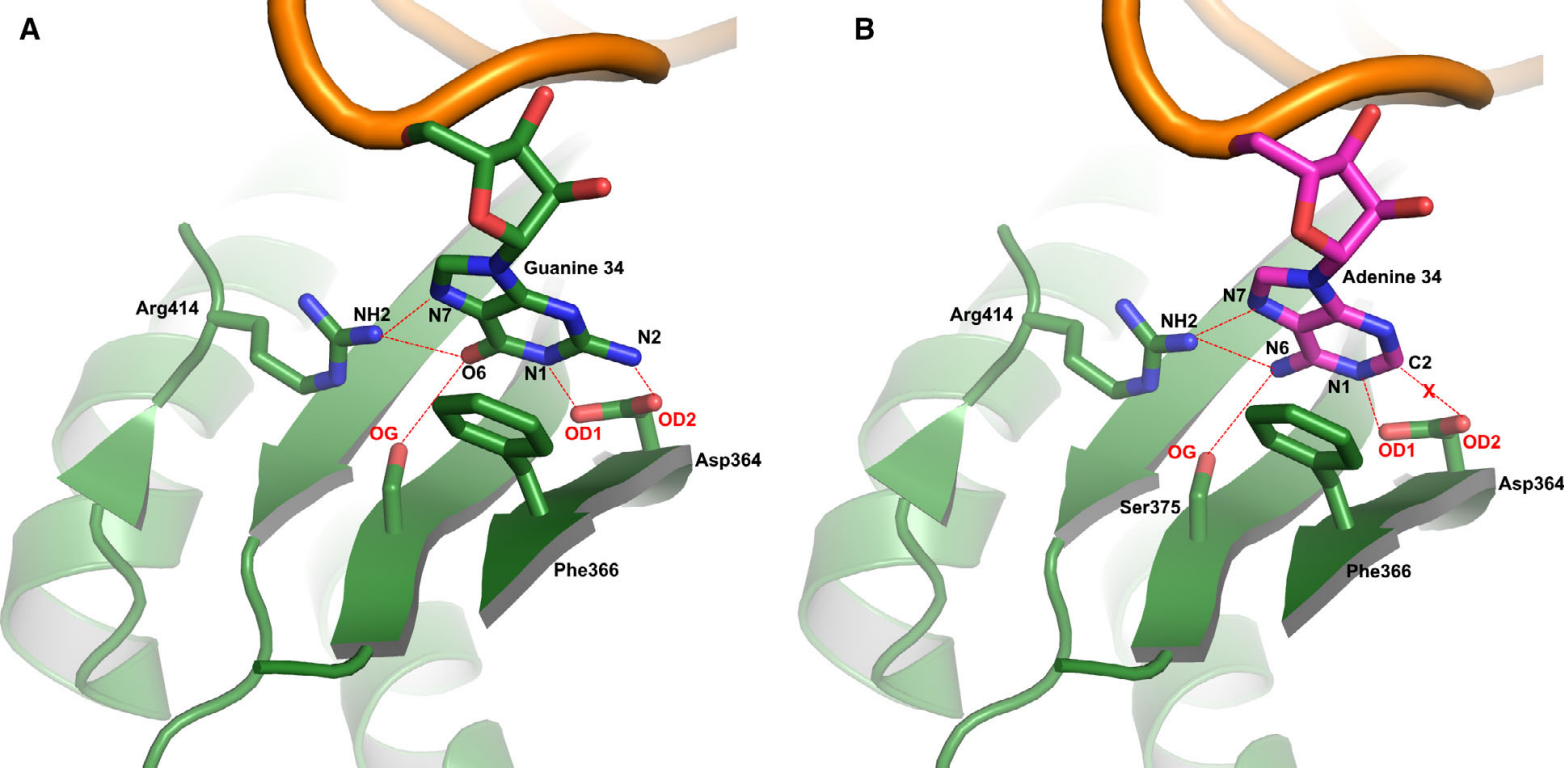
Primary recognition of Guanine‐34 from anticodon triplet of tRNA^Phe^ by ABD of *hmit*‐PheRS. (A) The contacts of G34 with key amino acids from ABD as revealed by the crystal structure of *hmit*‐PheRS complexed with tRNA^Phe^; (B) the modeled contacts of mutant A34 with ABD of *hmit*‐PheRS. Hydrogen bonding depicted by the dashed lines. Panels were prepared using program pymol [49].

Comprehensive analysis of the existing aaRSs‐tRNA crystal structures showed that the majority of anticodon tRNA guanine (G) interactions in various aaRSs‐tRNAs complexes specifically use it as an important tRNA identity element [26, 27]. This has relevance also from the crystal structure of human mitochondrial Phenylalanyl‐tRNA synthetase (*hmit*‐PheRS) complexed with tRNA^Phe^. The *hmit*‐PheRS consists of three structural domains: a unique N‐terminal domain, a class II catalytic domain (CD), and ABD (Fig. 1) [28, 29, 30]. The extensive net of stacking and hydrogen bonding intermolecular interactions is formed specifically by Guanine‐34 (G34) with the anticodon binding domain (ABD) of *hmit*‐PheRS [28, 31, 32]. Moreover, mode for stereo‐chemical recognition of G34, belonging to the anticodon triplet, is not unique among other aaRSs, and it had been imbedded through a process of convergent evolution. This format for selecting G, in many way similar to each other, must therefore be robust [27]. The primary recognition of tRNA^Phe^ by *hmit*‐PheRS is accomplished via an interaction of G34 with Phe366 (equivalent to Tyrβ731 in *Tt*‐PheRS) and with Asp364, Ser375, and Arg414 (Fig. 2A) [28, 32]. Stacking interaction between G34 and the phenyl ring of Phe366 is complemented by the formation of hydrogen bonds between the carboxylic groups of the Asp364 with N1 and N2 atoms of G34 (Fig. 2A). Moreover, Ser375 also establishes a H‐bond with O6 of G34 (3.2 Å). The hydrogen bond between the N7 atom of G34 and the guanidinium group of Arg414 contributes to further stabilization of the anticodon loop interacting with ABD. The ABD residues Asp364, Arg414, and Ser 375 belonging to *hmit*‐PheRS are either invariant or well conserved in ABDs of various bacterial PheRSs. Moreover, a stacking interaction between benzene ring of Phe366 (or its analog in other aaRSs) and guanine base, coupled with the net of hydrogen bonding interactions formed by aaRSs residues with N1, N2, N7, and O6 atoms of G34, appears to be a common mechanism for recognizing anticodon guanine [27]. The contribution of A‐35 and A36 from the anticodon triplet to recognition tRNA^Phe^ by *hmit‐*PheRS is insignificant, and they are considered as minor determinants for aminoacylation tRNA^Phe^ [28, 32]. It is, therefore, very interesting that a naturally occurring of G34A point mutation in *hmit*‐tRNA^Phe^ has been identified as causative for the mitochondrial condition, myoclonic epilepsy with ragged red fibers (MERRF) syndrome [33]. The severe loss in aminoacylation efficiency provides evidence that G34 serves as an identity element for *hmit*‐PheRS [34]. A defect in decoding of phenylalanine triplets can also arise from a dysfunctional Phenylalanyl‐tRNA synthetase mitochondrial protein coding gene (FARS2), encoded by *hmit*‐PheRS, and indeed, a 9‐year‐old female patient harboring a p.Asp364Gly mutation in the FARS2 gene has been reported recently. A girl born to consanguineous South Asian parents whom presented with a progressive spastic paraparesis and developed increasing bulbar difficulties affecting speech and feeding on her teenage years.

Here, we present the in‐depth analysis of changes induced by various mutations in the ABD area of *hmit*‐PheRS that are involved in primary recognition of tRNA^Phe^ anticodon loop. We have measured rates of tRNA^Phe^ aminoacylation by WT *hmit*‐PheRS and four mutant versions. These data provide evidence that specific nucleotides in the tRNA and amino acid residues within the *hmit*‐PheRS are absolutely crucial for the signal transduction pathway in the aaRSs‐tRNA complex. Although X‐ray structures of such complexes provide very important information regarding the hydrogen bonding network and salt bridges at tRNA–aaRSs interfaces, the inclusion of molecular dynamic (MD) correlations provides a more detailed picture of the network topology and possible deviations from time‐averaged intermolecular pictures. In combination with high‐resolution crystal structures of wild‐type *hmit*‐PheRS alone and complexed with *mit*‐tRNA^Phe^ [27, 28], we have performed extensive MD simulations to reveal the inter‐residue contact topology on the dynamic trajectories of *hmit*‐PheRS, caused by the reported point mutations. Furthermore, we have analyzed the dynamic behavior of residue–residue communication networks in the presence and absence of the tRNA^Phe^.

## Results

### Kinetic study of functional activities of *Hmit*‐PheRS mutants

**Fig. 3:**
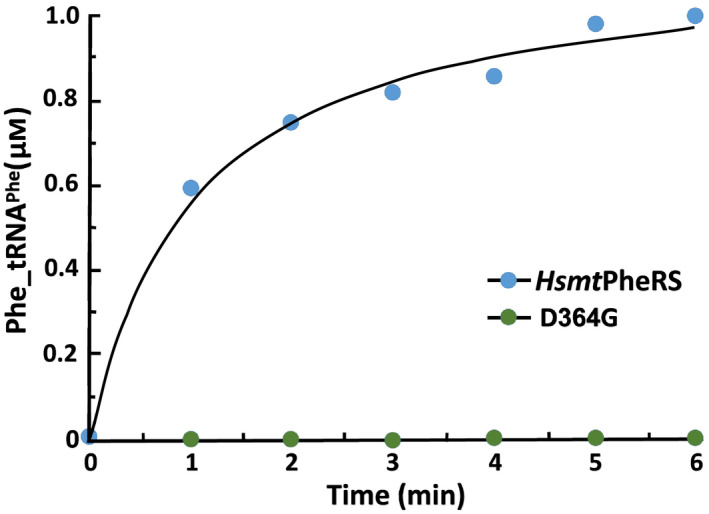
Phe‐tRNA^Phe^ formation by wild‐type *hmit*‐PheRS and its mutant forms. Aminoacylation activity was measured by incorporation of [^14^C]Phe into tRNA. No detectable aminoacylation of tRNA^Phe^ with mutant Asp364Gly PheRSs could be seen in the experiment.

To establish the rules governing recognition between partner macromolecules *hmit*‐PheRS and tRNA^Phe^ in the ABD area, and to explore the effect of breaking the hydrogen bonds between G34 and amino acids in the environment of the ABD, we measured of the aminoacylation activity of the tRNA^Phe^ by WT *hmit*‐PheRS and its mutated variants (Fig. 3 and Table 1). To keep the dynamic characteristics and main‐chain flexibility of the *hmit*‐PheRS structural fragments that form hydrogen bonding with G34, the Ser375 and Arg414 residues have been replaced with Ala. Thus, the pathogenic mutant Asp364Gly and three specifically designed mutants Arg414Ala, Ser375Ala, and a double mutation (Arg414Ala, Ser375Ala) were used to reveal the molecular basis of the G34 recognition by *hmit*‐PheRS. Kinetic experiments showed a dramatically reduced activity of the mutated Asp364Gly variant: in fact, this *hmit*‐PheRS mutant did not show any detectable aminoacylation activity (Fig. 3A). Defects in aminoacylation of this mutant linked directly to a loss of hydrogen bonding interactions between two carboxyls of Asp364, and N1 and N2 atoms of G34 (Fig. 2B). The loss of these specific interactions makes the mutated variant Asp364Gly inefficient in tRNA^Phe^ recognition and leads to severe problems in formation nascent polypeptide chains. It is of interest that having only one stacking interaction between G34 and aromatic ring of the Phe366 does not guarantee the recognition of tRNA^Phe^ by *hmit*‐PheRS.

**Table 1:** Relative activity of Phe‐tRNA^Phe^ formation by wild‐type *hmit*‐PheRS and its mutant forms. Aminoacylation was measured by incorporation of [^14^C]Phe into tRNA^Phe^. Aminoacylation activity for WT *hmit*‐PheRS and its specifically designed mutated variants. N.D. (not detectable) aminoacylation of tRNA^Phe^ with mutant Asp364Gly PheRSs seen in experiments.

Enzyme		Relative activity (%)
W.T. *hmit*‐PheRS		100
*hmit*‐PheRS	S375A	71.6
*hmit*‐PheRS	R414A	31.5
*hmit*‐PheRS	S375A/R414A	6.0
*hmit*‐PheRS	D364G	N.D.

**Fig. 4:**
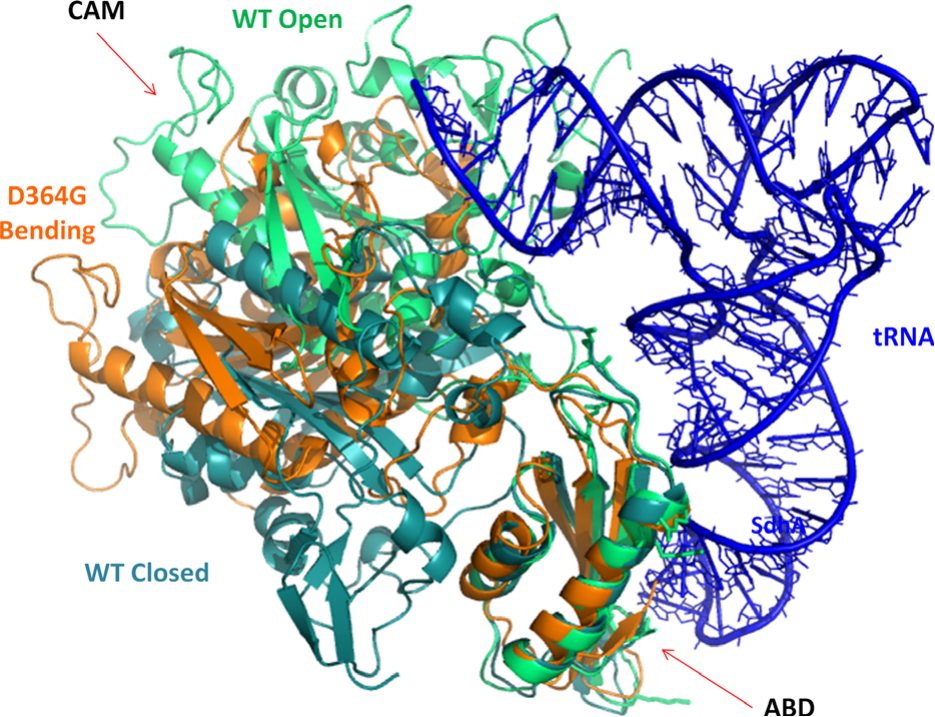
The MD simulations for the WT *hmit*‐PheRS and disease‐causing mutated variant Asp364Gly. To compare the result with the WT *hmit*‐PheRS dynamics, a superposition of three structures is shown: the WT open state (light‐green) and the ‘closed’ inactive conformation (deep‐green) of the WT protein; deep‐orange cartoon corresponds to the variant Asp364Gly on its pathway toward the ‘closed’ state. The 50‐ns snapshot of the 100‐ns dynamics trajectory of the Asp364Gly is shown. This conformation of the CAM and ABD looks as the predominant mode of the mutant's biased/altered dynamic behavior, thereby reducing the efficiency of tRNA aminoacylation that occurs in the open active conformation (green). Panels were prepared using program pymol [49].

Kinetic characteristics of the three mutated enzymes are shown in Fig. 4. These data reveal that all three specifically designed *hmit*‐PheRS variants are capable of catalyzing the aminoacylation of its tRNA^Phe^, albeit with significant variations in their efficiency. The resulting functional activity of the Ser375Ala mutant turned out to be similar to the activity of the WT protein (Table 1). The reason is that the polar Ser375, via the OG atom, establishes only one hydrogen bond (among the five hydrogen bonds stabilizing the position of G34) with O6 of G34 (3.2 Å), so structural stability, as well as recognition of tRNA^Phe^ by alanine‐substituted analog, was relatively affected. The Arg414Ala mutant displayed considerable reduction of the aminoacylation activity as compared to WT and Ser375Ala variants. The guanidinium group NH1 of Arg414 forms two hydrogen bonds with O6 and N7 of G34. Further, the availability of two hydrogen bonds between the side chain of Arg414 and G34 confers an important role on this residue, which contributes significantly to the recognition process and stabilization of the contacts between ABD and anticodon loop of tRNA^Phe^. It is clear from the acquired kinetic data, however, that the contribution of Asp364 to the recognition of the anticodon loop of tRNA^Phe^ by ABD of *hmit*‐PheRS, and to the formation of a stable complex proved to be of critical importance, when compared to the double mutant (S375A/R414A), or any other mutation leading to disappearance of hydrogen bonding with O6 and N7 atoms of G34.

### Molecular dynamic simulation of the wild‐type *hmit*‐PheRS and its mutants

As mentioned above, mutations in *mit*‐aaRSs can frequently be phenotypically recognized, such that clinicians can specifically identify the affected enzyme. Impaired aminoacylation activity of various mutated aaRSs has been demonstrated experimentally [9]. The expansion in exome and whole‐genome sequencing has identified an increasing number of mutations in *mit*‐aaRSs. Such mutations have been found also in the ABD of *hmit*‐PheRS. These may be considered as crucial since they result in the disturbance of primary recognition of the tRNA anticodon loop and, as a consequence, cause a severe loss of aminoacylation efficiency in the active site of aaRSs, where the 3′ CCA terminus of tRNAs should be located. The separation of the G34 (anticodon loop) and A76 (CCA at the 3′‐end of tRNA^Phe^) in *hmit*‐PheRS is ~ 80 Å. Thus, it is essential to investigate perturbations in this region to gain insight into the signal transduction from the anticodon loop to the active site area of aaRS and to examine precisely how mutations affect this process. Considering the dynamic characteristics of *hmit*‐PheRS [28, 29], when ABD and CD proceed through the ‘open’ and ‘closed’ conformations of induced fit with tRNA^Phe^ to give rise the productive complex, the trajectories of mutual adaptations of mutated variants of *hmit*‐PheRS and tRNA are not apparent even in the presence of the WT *hmit*‐PheRS complexed with tRNA coordinates at atomic resolution. The X‐ray coordinates typically provide averaged in‐time and ensemble‐averaged snapshots of the dynamic process of *hmit*‐PheRS–tRNA^Phe^ complex formation. In the case of *hmit*‐PheRS–tRNA complex, the MD simulation gives insight into this longstanding problem and helps in understanding the role of mutations in the relative displacement of ABD and the CD upon tRNA binding. Thus, using this method we will be able to follow the time‐dependent pathways from inactive ‘closed’ to active ‘open’ conformations and back, for WT *hmit*‐PheRS and the mutant forms. This will permit an appraisal of the stability thresholds for productive *hmit*‐PheRS–tRNA^Phe^ complex formation.

We performed the 100‐ns MD simulations for both the WT *hmit*‐PheRS and mutated Asp364Gly variant. Snapshots for the MD of Asp364Gly mutant stopped at 50 ns on the pathway trajectory, since after ~ 20 ns the CD undergoes rotation toward the ‘closed’ inactive conformation (Fig. 4, deep‐green) of the WT protein, and no longer changes a given state. In the dynamic two‐domain system (CD and ABD), the hinge type rotation occurs around residues Ser322‐Lys323. The transition from a ‘closed’ to an ‘open’ conformation in this case may be presented as a rotation of the ABD around CD, or the CD around ABD. The second type of rotation is presented in Fig. 4, demonstrating that the CCA‐end of tRNA^Phe^ is emerging from the active site of the Asp364Gly mutant. Such a mutual arrangement of the CD and ABD looks to be the predominant dynamic behavior (Fig. 4, conformation in brown) of the mutant, thereby reducing the efficiency of tRNA aminoacylation. A similar dynamic behavior has been observed in the Pro49Ala mutant of *hmit*‐PheRS: Its ABD and CD preferably demonstrate ‘closed’ configuration less accessible for tRNA binding [9]. It is of note that mutations in different domains ABD and CD induce dynamically similar states in the protein conformational space.

Insight into the dynamic relationship between CD, ABD, and tRNA^Phe^ and two functionally different forms of *hmit*‐PheRS (free and complexed with tRNA) can be attained by the dynamic cross‐correlation maps using elastic network models (ENMs) [35, 36, 37]. The stereo‐chemical conformations and flexibility of both proteins and tRNAs are completely defined by covalent and hydrogen bonds. These constraints can be modeled as linear spring connections between spatially proximal representatives in a variety of coarse‐grained ENMs. Thus, the two structural domains CD and ABD together with tRNA are represented by an elastic network of its components connected by springs. This approach makes it possible to draw conclusions about the stability of both free *hmit*‐PheRS and that complexed with substrate tRNA.

**Fig. 5:**
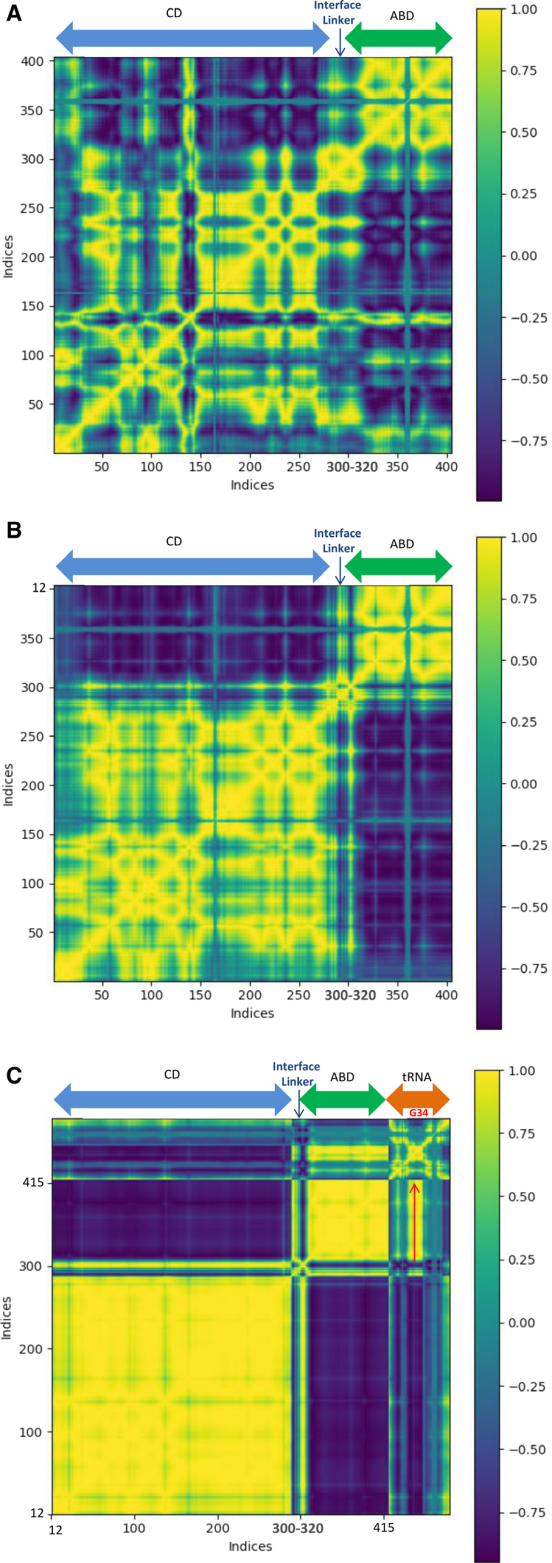
Dynamic cross‐correlation maps calculated using MD/ENM methods for PheRS‐tRNA residues and nucleotides. Strong residue–residue corresponds to +1 (yellow), while regions of anticorrelated (−1) fluctuations are colored dark blue. The numbers along horizontal and vertical axes correspond to PheRS (12‐415, panels A, B, C). The structural and dynamics integrity within the CD (light blue arrow) and the ABDs (green arrow) increase from a closed, ‘non‐active’ conformation of *hmit*‐PheRS (A) to the open ‘active’ state (B), and reaches its maximum, making complex with tRNA (orange arrow, C).

The MD calculations presented in Fig. 5 clearly illustrate that the mean intradomain correlation of atomic motions serves as a quantitative criterion of the domain structural independence and dynamic integrity. At the same time, the CD (light blue arrow) and ABD (green arrow) modules that possess a high level of internal stability and are covalently connected by an extended polypeptide segment demonstrate functional integrity and strong interdependence. Dynamic transition from the closed ‘inactive’ (panel A) to the open ‘active’ conformation (panel B) is accompanied by an increase in the internal stability of CD and ABD modules. This is most clearly manifested in the formation of a complex between *hmit*‐PheRS with its cognate tRNA^Phe^ (orange arrow, panel C).

The appearance of less organized and diffuse patterns seen in Fig. 5 (panel A) suggests the presence of many cross‐correlated low‐amplitude fluctuations in the CD/ABD *hmit*‐PheRS molecule. It is reasonable to assume that a given representation is the consequence of the large number of contacts between CD and ABD in the inactive ‘closed’ conformation (panel A). In the extended open ‘active’ conformation (Fig. 5, panel B), the CD and ABD modules, connected by flexible interdomain polypeptide linker, rotate around the hinge joint and move along uncorrelated independent trajectories. The *hmit*‐PheRS dynamic behavior may be considered as a movement of two rigid‐body modules (CD and ABD), around a short polypeptide stretch integral to this region. Two large yellow squares representing domains CD (12–310) and ABD (324–415) are clearly visible on Fig. 5 (panel B). Fluctuations in the 300–324 (panel B) correlate with the dynamics of the CD/ABD domains. Moreover, detailed analysis of the MD/ENM data for the first three slowest fluctuation modes has identified residues 315–319 as a hinge region. Therefore, the region 311–323 serves as a dynamic interface between CD and ABD.

The MD calculations for the *hmit*‐PheRS–tRNA^Phe^ complex represent a very simple and pronounced modular pattern (Fig. 5C). The tRNA^Phe^ anticodon region centered on G34 (dense region indicated by red arrow) forms an extensive network of dynamic cross‐talk interactions between charged residues located at the CD/ABD interface region. There is exceptionally high mobility of the structural domains CD and ABD in *hmit‐*PheRS, which is not common for other aaRSs [28, 29, 38]. Despite this mobility, upon binding tRNA^Phe^ the complex demonstrates remarkable simplicity in the cross‐correlation map (Fig. 5C). The fluctuations visible in panels A and B (Fig. 5) turned out to be very small, practically indistinguishable (panel C), and the majority of the hinge sites/residues disappear in this map. Thus, due to the overall stabilization of the complex the cross‐correlation map demonstrates remarkable simplicity of its dynamic characteristics.

Based on ENM calculation, it is reasonable to conclude that Asp364 appears as a hinge residue, in fast fluctuation modes of the ‘active’ *hmit*‐PheRS conformation when the protein has still not complexed with tRNA. Indeed, data analysis of MD trajectories confirms that the surface residue Asp364 is significantly less flexible than the Arg414 or even the Ser375 located inside of the ABD.

### Vibrational analysis: interface backbone flexibility of PheRS ABD

Applying MD simulations to the wild‐type *hmit*‐PheRS and its mutated variants, we have revealed important conformational characteristics and intramolecular interactions within the ABD, a domain responsible for primary recognition of the tRNA^Phe^ and subsequent signal transmission to the active site, to start the aminoacylation reaction. Data analysis and interpretation of series of snapshots led us to the conclusion that wild‐type *hmit*‐PheRS and those mutants that demonstrate a comparatively high level of aminoacylation activity retain the open state conformation, as has been observed for in complex with tRNA. These conclusions are strongly supported by small‐angle X‐ray scattering (SAXS) data [29]. SAXS experiments were carried out on the WT enzyme complexed with tRNA^Phe^, and ‘inactive’ variant, when K33 and T351 were substituted with cysteines, thereby generating the ‘inactive’ conformation of *hmit*‐PheRS [29].

**Fig. 6:**
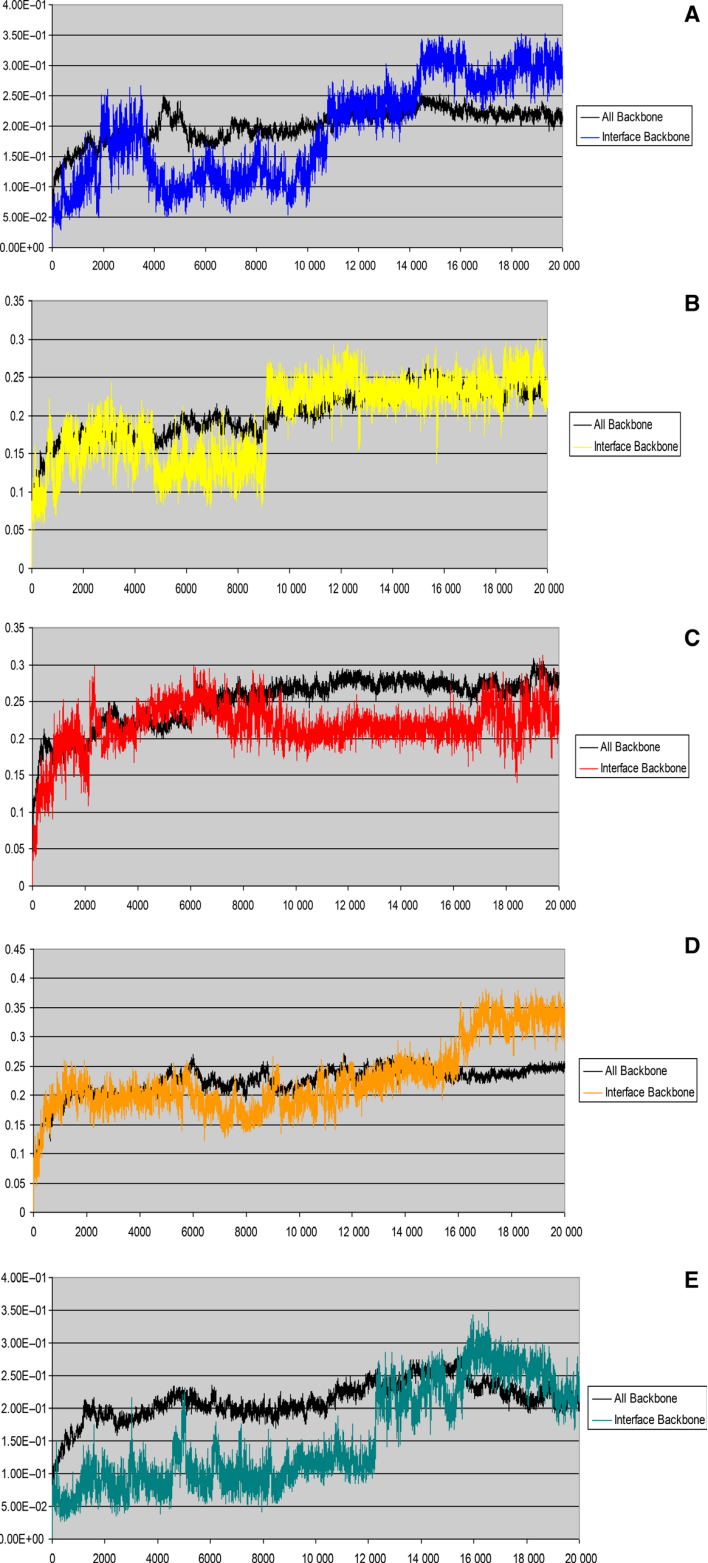
The oscillation patterns of atoms located at the interface between ABD of *hmit*‐PheRS and tRNA^Phe^. Presented are the rmsd fluctuations of backbone atoms belonging to the residues exposed to the interface vs. all backbone atoms of ABD; (A) ABD of WT *hmit*‐PheRS; (B) ABD of Ser375Ala mutant *hmit*‐PheRS; (C) ABD of Arg414Ala and Ser375Ala double‐mutant *hmit*‐PheRS; (D) ABD of Asp364Gly mutant *hmit*‐PheRS; (E) For comparison, we present the oscillation pattern for a mutation Asp289Tyr [9] located within amino acid sequence between CD and ABD.

MD contact patterns at the interface between anticodon loop of tRNA^Phe^ and ABD of *hmit*‐PheRS reveal highlights of the natural motion of the molecular binary system, and can be used as a complementary tool for the analysis of both experimental and dynamic structures with a selectively modified interface. These allow us to monitor the relaxation process when atoms are moving from their original positions. Oscillation patterns suggest (Fig. 6) the behavior of two sets of backbone atoms belonging to the ABD as a whole, and these residues that exposed to the interface, differ significantly from each other. The initial conformation of the interface formed by tRNA^Phe^ bound to *hmit*‐PheRS was taken as the reference point. The graphs display rmsd of the atoms located in this area that refer to WT *hmit*‐PheRS ABD (Fig. 6A) and mutants: Ser375Ala (Fig. 6B), double‐mutant Arg414Ala and Ser375Ala (Fig. 6C), and Asp364Gly (Fig. 6D) along the first 10 ns of the simulation. As a control, we also presented the mutant Asp289Tyr (Fig. 6E). Residue Asp289 is located at the interface of CD and ABD, and at a distance from the synthetic active site and the interface between ABD and anticodon loop of tRNA^Phe^ [9]. The curves were obtained after averaging several MD trajectories for each mutated variant, and conformational stability of the bound‐like state was defined as the percentage of simulation time during which the backbone atoms of ABD exposed to the interface remain close to those observed in tRNA‐bound conformation (within 0.15 nm rmsd). The WT PheRS ABD visits the bound‐like state conformation more frequently than that associated with Asp364Gly and Arg414Ala mutants. The MD demonstrated that the WT is trapped in the bound‐like conformation for a significantly longer period of time as compared to those in mutated variants. Time evolution of the ABD interface backbone conformation (during first 20‐ns window of 100‐ns MD) reveals the transition from bound‐like to an unbound (relaxed) state. As can be seen in the Fig. 6A, the irreversible transition, that is, the ‘distortion’ of the starting conformation of the WT protein, occurs in ~ 11 ns, such that during this period, the interface area fluctuates around the initial conformation, deviating from it on average no more than 1.5 Å. As a control, we measured the interval of conformational transitions for mutant Asp289Tyr [9] that is not exposed to the interface area. The relaxation time for Asp289Tyr was ~ 10 ns. The irreversible transition for mutant Ser375Ala occurs in a relatively shorter period of time – 8 ns Fig. 6B. In the case of a specifically induced double‐mutant Arg414Ala and Ser375Ala Fig. 6C and the disease‐causing mutation Asp364Gly [39] Fig. 6D, the interface area goes into irreversibly relaxed state very quickly during 1–2 ns.

## Discussion

Based on the fast kinetic experiments, fluorescence titrations, and ultracentrifugation analysis, Krauss *et al*. [40] hypothesized that binding of tRNA by aaRSs proceeds in two steps. The initial bimolecular step is rapid and has a broad specificity, whereas the second unimolecular step is related to conformational changes and more precise adjustment and/or recognition. Broad specificity is associated with electrostatic interactions that are considered as the major driving forces upon the formation of any aaRS‐tRNA complex, and navigate tRNA toward the ABD of aaRS [40, 41, 42]. The second unimolecular step is related to conformational changes and more precise adjustment/recognition of tRNA. It is the second stage that has a decisive influence on the functional characteristics of aaRS, that is, on the efficacy of aminoacylation. Since most aaRSs, upon tRNA binding, recognize specifically only few nucleotides, it is understandable that mutations of nucleotides belonging to the anticodon triplet are tolerated less well, strongly affecting aminoacylation activity, and so are less common [12, 34]. A non‐synonymous replacement of the key nucleotide in this triplet prevents the formation of the correct net of hydrogen bonds and stacking interactions, thereby reducing or eliminating the primary recognition of a mutant tRNA by the corresponding aaRS. The majority of class II aaRSs approach the tRNA anticodon loop from the major‐groove side, and the anticodon bases have to protrude out to form base‐specific contacts with aaRS [43]. By contrast in PheRS, also structurally belonging to class II [44, 45], such contacts exist on the minor groove side of the anticodon loop, and bacterial tRNA^Phe^ keeps the conformation of the anticodon loop relatively similar to that of free tRNA^Phe^, thereby maintaining its almost undistorted conformation [32]. Ling *et al*. [34] investigated the impact of the pathogenic mutation G34A on both global tRNA structure and different steps in translation. A significant observation that has been interpreted by the authors is that the impact of the G34A anticodon mutation does not affect global folding of tRNA^Phe^, and displays the same migration pattern as the WT G34 tRNA^Phe^, thereby indicating that appearance of adenine instead of guanine at the anticodon triplet does not change the 3D structure of tRNA^Phe^. From this, we can conclude that the adenine base will be associated with the benzene ring of phenylalanine by a stacking interaction, and further connected with the amino acid residues of *hmit*‐PheRS capable to the formation of the hydrogen bonding net in the same way as guanine. These two interaction modes are suggested to be responsible for the primary recognition of both WT G34 and mutated A34 version of tRNA^Phe^ by *hmit*‐PheRS. Analysis of mutant‐dependent relaxation processes indicates that mutations present in residues exposed to the interface will destabilize the formation of primary recognition of the anticodon loop of tRNA, and subsequent signal transduction to the active site.

Depending on their location in the polypeptide chain of *hmit*‐PheRS, some mutations can minimize aminoacylation activity, as happens with the double‐mutant Arg414Ala and Ser375Ala, or completely inactivate the enzyme, as is the case with Asp364Gly (Fig. 3). In contrast, the pathogenic G34A mutation within the anticodon loop of the substrate tRNA^Phe^, and associated with the MERRF syndrome, decreases the aminoacylation activity of *hmit*‐PheRS by 100‐fold (Fig. 2B) [34]. This mutation is accompanied by the loss of one hydrogen bond between OD2 atom of Asp364 and C2 atom of the adenine base. The Asp364Gly mutation in the *hmit*‐PheRS results in the loss of two hydrogen bonds between Gly364 and G34 (Fig. 2A). Thus, we may conclude that the starting conformation of the disease‐causing mutant, Asp364Gly, is the least stable. The specifically induced double‐mutant Arg414Ala and Ser375Ala, which breaks three hydrogen bonding contacts (Fig. 2) with G34 (or with A34 in the mutated tRNA^Phe^ [34], also contribute significantly to the destabilization of the initial bound‐state conformation. For comparison (and control), the Tyr289 mutation is also shown, which does not lead to very fast destabilization (Fig. 6E). In this case, the relaxation time (12 ns) is almost the same as in the native protein. Accordingly, we may conclude that Asp364 belongs to the small set of the residues providing the precise adjustment of *hmit‐*PheRS and tRNA^Phe^ complex formation, and replacing it with another amino acid may be critical for normal functioning of the enzyme.

By analyzing such a complex network of tRNA recognition by aaRSs, we come to the conclusion that loss of a one hydrogen bond in tRNA^Phe^ anticodon recognition by Asp364Gly mutant as compared to the G34A mutation in *mit*‐tRNA^Phe^ may lead not only to a complete inactivation of the *hmit*‐PheRS, but also to a different disease phenotype. Thus, mutations of hydrogen bonding between OD1 and OD2 carboxyls of Asp364 from *hmit‐*PheRS, and N1 and N2 of G34 from substrate tRNA^Phe^ side at the WT *hmit*‐PheRS, may cause pleiotropic effects. This suggests that a single molecular function of primary mitochondrial tRNA^Phe^ recognition by *hmit*‐PheRS may have a consequence on multiple biological processes.

## Materials and methods

### Construction of plasmids for expression of *hmit*‐PheRS mutants

The gene encoding wild‐type *hmit*‐PheRS cloned in pET21a (Novagen, Madison, WI, USA) was used as a template. The plasmids encoding the mutant variants were prepared using the Transfer‐PCR method https://www.ncbi.nlm.nih.gov/pubmed/24395359).

The primers used to generate mutants are listed below, with the exchanged bases underlined. S375A mutation used *hmit*‐PheRS_S375A_F (5′‐CATCCAAAGACGCACAAGACCGCTCACTGCTACCGCATCACGTAC) and PetRev (5′‐ ATGCTAGTTATTGCTCAGCGGT). R414A mutation used *hmit*‐PheRS_R414A_R (5′‐ GGTGGTGCTCGAGTCATCAGAAAGCGCCCTCCACACCCAACAGCTG) and T7 (5′‐ ATTAATACGACTCACTATAGGGG). For generation of the double mutant (S375A/ R414A), primers *hmit*‐PheRS_S375A_F and *hmit*‐PheRS_R414A_R (described above) were used. The mutated plasmid D364G was prepared with the Stratagene QuikChange mutagenesis kit (Agilent Technology, Santa Clara, CA, USA), according to the manufacturer’s instructions, using two complementary primers. The next primers were used to generate *hmit*‐PheRS‐D364G F (5′ CCTGGAAAAGGTTGATCTCATAGGCAAGTTTGTACATCCAAAGACGC) and I (5′ GCGTCTTTGGATGTACAAACTTGCCTATGAGATCAACCTTTTCCAGG).

### Purification of the mutated mitPheRS

*Escherichia coli* Rosetta DE3 strain cells (Novagen) were transformed with the various plasmids and cultured at 37° C in 1 L batches of LB medium supplemented with 100 µg·mL^−1^ ampicillin to an OD of 0.6 at 600 nm. The cells were then induced with 1 mm IPTG and cultured overnight at 37° C. Cells were harvested by centrifugation at 3000 ***g*** for 30 min, disrupted with a French Press, and then centrifuged (100 000 ***g*** for 30 min) to remove cell debris. Enzymes were purified by chromatography on a 5 mL HiTrap heparin‐affinity column (25 × 16 mm; GE Healthcare, Marlborough, MA, USA), followed by a 600 × 16 mm size‐exclusion HiLoad Superdex 200 column (GE Healthcare). Purified samples were concentrated and dialyzed against buffer (20 mm Tris/HCl pH 8, 100 mm NaCl, 7 mm MgCl_2_, 5 mm 2‐mercaptoethanol, and 1 mm EDTA). Purified protein was stored in small aliquots and flash‐frozen at 193ºK.

### The tRNA aminoacylation assay

The activity of the mutated *hmit*‐PheRS variants (S375A, R414A, and S375A‐R414A) was tested in reaction mixtures containing 30 mm Tris/HCl, pH 8.5, 15 mm MgCl_2_, 5 mm ATP, 40 mm 2‐mercaptoethanol, 4 µm L‐[3 H]Phe, 4 µg·µL^−1^ mix *E. coli* tRNA, and 0.1 µg·µL^−1^ of mutated *hmit*‐PheRS protein. At the appropriate times, aliquots (5 µL) were spotted onto Whatman filter paper impregnated with 5% trichloroacetic acid (TCA). Then, the filters were extensively washed with 5% TCA, and TCA‐insoluble radioactivity was measured by liquid scintillation counting.

### Computational methods: MD and elastic network model

The WT *hmit*‐PheRS and all mutants were subjected to MD simulations (as described in [9]) with a limited ~ 100 ns. MD trajectories generated by computational protocols have revealed a series of conformational changes and global (interdomain) motions/fluctuations. It should be noted that the 100‐ns time range used for the ~ 400 amino acid protein like *hmit*‐PheRS does not guarantee that all available conformations were found. The conformational space of most macromolecules studied today is incompletely sampled due to computational feasibility, which in turn limits the ability to analyze and reveal the functional properties of the systems being examined [46].

The ENM is a collection of methods for sampling and analysis of correlated and anticorrelated, thermally accessible fluctuations in macromolecules [35]. The ENM methods based on statistical mechanics enables the representation of global and local molecular motions as a spectrum of slow and fast modes. In this study, we have used the Gaussian network model (GNM), which is the simplified ENM assuming that all fluctuations are isotropic [35, 36]. Pairwise distances in space between structural units (amino acids and nucleotides) are approximated by the Kirchhoff adjacency matrix, and nonbonded interactions are defined by an appropriate single‐parameter harmonic potential [37].

The ProDy open‐source Python package [47] was used in GNM calculations. We have applied a protocol similar to that used for tRNA molecules both in free and synthetase‐bound states [48]. The *hmit*‐PheRS Cα and tRNA phosphate atoms were used to construct Kirchhoff matrices and to build contact maps, with the cutoff distance of 20 Å applied to the PheRS structure (PDB code 3CMQ) and the PheRS‐tRNA complex (PDB code 3TUP). The cross‐correlation maps were build using ProDy and Matplotlib python libraries.

## Conflict of interest

The authors declare no conflict of interest.

## Author contribution

MP performed biological, biochemical and kinetic experiments. DT did MD calculations and wrote the paper. EK performed biochemical and kinetic experiments. JL provides and analyzed clinical data. ZCL provided and analyzed clinical data and wrote the paper. MS formulated problem, supervised the work, and wrote the paper.
